# Mantle Cell Lymphoma with Persistent Massive Pleural Effusions Requiring Invasive Mechanical Ventilation and Bilateral Continuous Thoracic Drainage

**DOI:** 10.3390/reports9010038

**Published:** 2026-01-27

**Authors:** Taichiro Tokura, Youhei Imai, Satoshi Sakai, Reina Saga, Hiroko Hidai, Sayuri Motomura

**Affiliations:** 1 Department of Hematology, Tokyo Metropolitan Tama-Hokubu Medical Center, 1-7-1 Aoba-cho, Higashimurayama-shi, Tokyo 189-0002, Japan; 2 Department of Hematology, Nippon Medical School, 1-1-5 Sendagi, Bunkyo-ku, Tokyo 113-8602, Japan

**Keywords:** mantle cell lymphoma (MCL), pleural effusions, endotracheal intubation, invasive mechanical ventilation, chest tube, thoracic drainage, respiratory failure, critical care, immunochemotherapy, malignant lymphoma

## Abstract

**Background and Clinical Significance:** Mantle cell lymphoma (MCL) frequently involves bone marrow, gastrointestinal tract, and hepatosplenomegaly, whereas pleural effusions are uncommon. Cases requiring invasive mechanical ventilation and thoracic drainage are rare. We report a case of MCL with persistent massive pleural effusions requiring invasive mechanical ventilation and bilateral continuous thoracic drainage. **Case Presentation:** A 71-year-old woman presented with dyspnea and was found to have bilateral pleural effusions and generalized lymphadenopathy. Shortly after admission, she developed acute respiratory failure due to pleural effusions and required invasive mechanical ventilation. Right-sided continuous thoracic drainage was initiated. Thereafter, more than 1 L of pleural fluid was drained each day. Flow cytometry of the pleural fluid showed CD5-positive B cells with kappa light-chain restriction. Bone marrow examination revealed abnormal lymphocyte infiltration. Cervical lymph node biopsy demonstrated diffuse proliferation of medium-sized, abnormal B lymphocytes with an immunophenotype of CD5^+^, CD19^+^, CD20^+^, cyclin D1^+^, SOX11^+^, and κ^+^, with a Ki-67 index of 20%, confirming MCL, stage IV. Immunochemotherapy with rituximab, cyclophosphamide, doxorubicin, vincristine, and prednisone (R-CHOP) was commenced under mechanical ventilation. Shortly thereafter, left-sided continuous thoracic drainage was also initiated. However, in response to immunochemotherapy, the bilateral pleural effusions gradually subsided, enabling extubation, and there was no reaccumulation after removal of both chest tubes. Furthermore, generalized lymphadenopathy regressed, and bone marrow examination revealed resolution of lymphoma infiltration, resulting in complete remission. **Conclusions:** De novo MCL complicated by persistent massive pleural effusions requiring invasive mechanical ventilation and bilateral continuous thoracic drainage is rare. A thorough diagnostic workup followed by prompt initiation of immunochemotherapy can arrest pleural output, enable extubation, and be lifesaving. Clinicians should recognize that MCL rarely presents with persistent massive pleural effusions.

## 1. Introduction and Clinical Significance

Mantle cell lymphoma (MCL) is an uncommon mature B-cell neoplasm characterized by the proliferation of medium-sized lymphocytes [1,2]. The translocation t(11;14)(q13;q32) leading to constitutive overexpression of cyclin D1 is a pathognomonic feature [1,2]. Patients typically present with generalized lymphadenopathy and are classified as Ann Arbor/Lugano stage III or IV [3]. Extranodal involvement is observed in approximately 75–90% of patients, including infiltration of bone marrow (53–92%), gastrointestinal tract (88%), peripheral blood (34–50%), spleen (27%), and liver (8–25%) [4,5,6,7]. However, pleural involvement and pleural effusions are rare, and reports of these as initial presenting symptoms are even fewer [8].

Clinically, MCL exhibits heterogeneous behavior, ranging from aggressive disease to indolent forms that may not require treatment for years [4]. This heterogeneity is partly reflected by two major subtypes: the more common conventional MCL (cMCL) and the less frequent leukemic non-nodal MCL (nnMCL) [1,2]. Both subtypes are clearly distinct in molecular characteristics and clinical presentation. cMCL very frequently harbors complex karyotypes with several driver gene alterations (median 7 per case), simultaneously affecting multiple pathways [1]. It presents with generalized lymphadenopathy and follows a more aggressive course [9]. In contrast, nnMCL usually displays a simple karyotype, dominated by t(11;14), with fewer driver gene alterations (median <2 per case) [1,10]. It does not cause lymphadenopathy, frequently presents with lymphocytosis and splenomegaly, and generally exhibits an indolent course with superior outcomes [9].

Current treatment paradigms are not subtype-specific. For both subtypes, induction immunochemotherapy, such as R-CHOP (rituximab, cyclophosphamide, doxorubicin, vincristine, and prednisone) or BR (bendamustine and rituximab), is recommended for symptomatic stage III or IV [4]. In recent years, the addition of Bruton tyrosine kinase (BTK) inhibitors, such as ibrutinib and acalabrutinib, to R-CHOP or BR has further improved clinical outcomes [4]. In addition, in patients <65 years and otherwise fit, high-dose consolidation therapy followed by autologous hematopoietic stem cell transplantation is the current standard of treatment [4]. Across subtypes, pleural involvement and pleural effusions are rare, and the need for invasive mechanical ventilation or continuous thoracic drainage during treatment is extremely rare. In particular, there are very few cases of de novo MCL requiring these interventions before diagnosis.

Here, we report a case of de novo MCL requiring invasive mechanical ventilation and bilateral continuous thoracic drainage due to life-threatening respiratory failure caused by persistent massive pleural effusions.

## 2. Case Presentation

A 71-year-old woman with no significant past medical history presented to a local hospital in September 2025, complaining of dyspnea. Chest-abdominal computed tomography (CT) revealed bilateral pleural effusions and lymphadenopathy in the cervical, mediastinal, and para-aortic regions. On 16 September, she was referred to our hospital for further evaluation and was admitted the same day. Physical examination revealed palpable right cervical lymphadenopathy and decreased breath sounds over the right lung field. No lower extremity edema or other significant abnormal findings were noted. Her vital signs on admission were: alert consciousness, body temperature 36.6 °C, heart rate 84 bpm, blood pressure 131/59 mmHg, respiratory rate 18/min, and SpO_2_ 95% on room air. Her Eastern Cooperative Oncology Group (ECOG) performance status was 2. The electrocardiogram showed no remarkable abnormalities. Laboratory tests showed a white blood cell count of 9300/μL (neutrophils 59.5%, lymphocytes 9.0%, monocytes 5.0%, and abnormal lymphocytes with indented nuclei and a high nuclear-to-cytoplasmic ratio 26.5%), hemoglobin 14.3 g/dL, platelets 29.7 × 10^3^/μL, lactate dehydrogenase (LDH) 278 U/L, C-reactive protein (CRP) 0.15 mg/dL, N-terminal pro-B-type natriuretic peptide (NT-proBNP) 207 pg/mL, and soluble IL-2 receptor (sIL-2R) 3730 U/mL, and hepatitis B and C virus serologies were negative.

**Figure 1:**
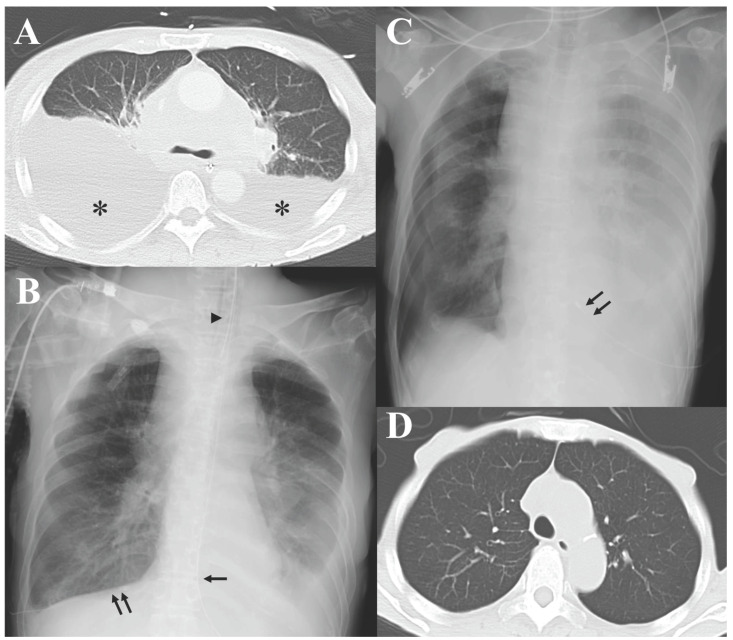
Radiological course of bilateral pleural effusions. (**A**): Chest computed tomography (CT) obtained immediately after endotracheal intubation, showing bilateral pleural effusions (asterisks), more prominent on the right side. (**B**): Chest X-ray during right-sided continuous thoracic drainage, showing a decrease in the right pleural effusion and persistent left pleural effusion; the arrowhead indicates the endotracheal tube, the arrow indicates the nasogastric tube, and the double arrows indicate the right chest tube. (**C**): Chest X-ray obtained after extubation and removal of the right chest tube, demonstrating progression of the left pleural effusion and the newly inserted left chest tube (double arrows). (**D**): Chest CT after immunochemotherapy and removal of both chest tubes, showing resolution of the bilateral pleural effusions without re-accumulation.

On the night of admission, she developed sudden respiratory failure, with her SpO_2_ dropping to 81%. Although supplemental oxygen was started, her oxygen saturation did not improve, so endotracheal intubation was performed, and invasive mechanical ventilation was initiated. Contrast-enhanced CT from the neck to the pelvis demonstrated lymphadenopathy in the bilateral cervical, supraclavicular, and hilar regions, as well as in the mediastinal, para-aortic, and mesenteric areas. Bilateral pleural effusions, more prominent on the right side, were also observed (Figure 1A). Transthoracic echocardiography showed preserved cardiac function with no remarkable abnormalities. Her acute respiratory failure was considered to be due to the pleural effusion, which worsened in the supine position. A chest tube was inserted into the right pleural cavity, and continuous thoracic drainage was initiated (Figure 1B). Although her respiratory status became stable, more than 1 L of pleural fluid continued to be drained each day thereafter.

**Figure 2:**
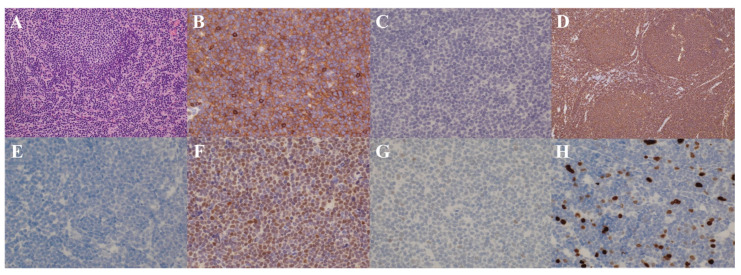
Right cervical lymph node biopsy showing diffuse proliferation of medium-sized, monomorphic abnormal B lymphocytes with an immunophenotype of CD5^+^, CD10^−^, CD19^+^, CD20^+^, CD23^−^, BCL2^+^, BCL6^+^, cyclin D1^+^, SOX11^+^, p53^−^, and κ^+^, with a Ki-67 index of approximately 20%. (**A**): Hematoxylin and eosin (×20). (**B**): CD5-positive (×40). (**C**): CD10-negative (×40). (**D**): CD20-positive (×10). (**E**): CD23-negative (×40). (**F**): cyclin D1-positive (×40). (**G**): p53-negative (×40). (**H**): Ki-67 (approximately 20% positivity, ×20).

Pleural fluid analysis showed a yellowish-brown appearance, a pH of 8.5, a nucleated cell count of 1109/µL with 34.5% abnormal lymphocytes, LDH 228 U/L, glucose 157 mg/dL, total protein 3.7 g/dL, albumin 2.6 g/dL, and a positive Rivalta test. Concurrent serum total protein and albumin levels were 5.9 g/dL and 3.5 g/dL, respectively, yielding a pleural fluid-to-serum protein ratio of 0.63, a serum-to-pleural fluid albumin gradient of 0.9 g/dL, and a serum-to-pleural fluid total protein gradient of 2.2 g/dL, consistent with an exudative effusion. Its culture was negative. Cytological examination revealed numerous large abnormal lymphocytes with irregular nuclei, classified as class V. Flow cytometry of the pleural fluid identified a cell population, slightly larger than the lymphocyte fraction, that was CD5^+^, CD19^+^, CD20^+^, and CD23^−^, with a κ/λ ratio of 23, suspicious for B-cell lymphoma. Bone marrow aspiration and biopsy revealed that abnormal lymphocytes with the same immunophenotype accounted for approximately 30% of the nucleated cells. On hospital day 9, an excisional biopsy of a right cervical lymph node was performed. As shown in Figure 2, histopathological examination of the lymph node demonstrated diffuse proliferation of medium-sized, monomorphic abnormal B lymphocytes with an immunophenotype of CD5^+^, CD10^−^, CD19^+^, CD20^+^, CD23^−^, BCL2^+^, BCL6^+^, cyclin D1^+^, SOX11^+^, p53^−^, and κ^+^, with a Ki-67 index of approximately 20%. Fluorescence in situ hybridization (FISH) for IgH/CCND1 on the lymph node specimen showed fusion signals in 94.0% of analyzed cells. Conventional karyotyping revealed a normal karyotype. The pleural fluid cell block and bone marrow specimens demonstrated similar findings. Taken together, these results established a diagnosis of MCL, stage IV. According to the MCL International Prognostic Index (MIPI), she was classified as high risk [11]. The Ki-67-based combined MIPI (MIPI-c) placed her as high-intermediate risk [12].

**Figure 3:**
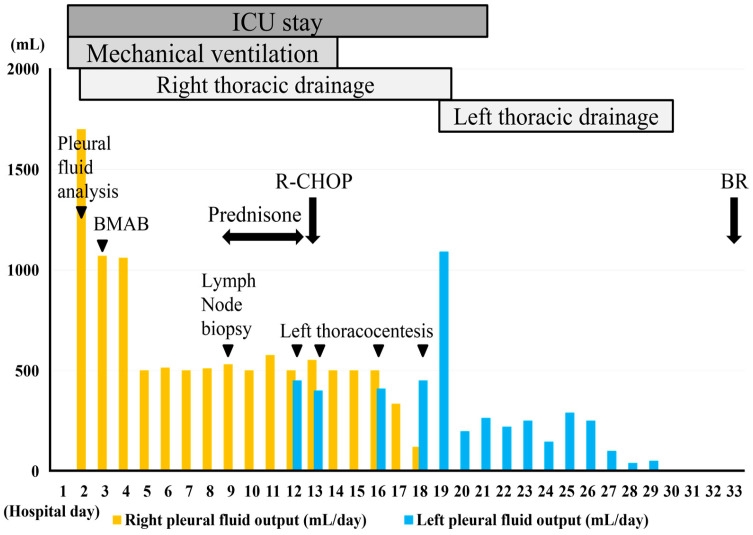
Clinical course of bilateral pleural fluid output and key events. Abbreviations: ICU, intensive care unit; BMAB, bone marrow aspiration and biopsy; R-CHOP, rituximab, cyclophosphamide, doxorubicin, vincristine, and prednisone; BR, bendamustine and rituximab.

On hospital day 9, following the lymph node biopsy, prephase therapy with prednisone 100 mg was initiated (Figure 3). Subsequently, her respiratory status gradually improved, and she was weaned from mechanical ventilation. On hospital day 13, because only CD20-positive B-cell lymphoma was suspected and definitive histopathology was still pending, and because her critical condition left no time to await the final diagnosis, the R-CHOP regimen was initiated, consisting of vincristine 1.5 mg (1.4 mg/m^2^), doxorubicin 53 mg (50 mg/m^2^), and cyclophosphamide 785 mg (750 mg/m^2^), each administered at 70% of the standard dose, with rituximab 560 mg (375 mg/m^2^). After initiation of R-CHOP, the right pleural effusion decreased, allowing removal of the right chest tube. A left-sided pleural effusion then required continuous thoracic drainage for a short time (Figure 1C), but the drainage output gradually decreased, and the left chest tube was also removed. From the second cycle onward, after MCL diagnosis was confirmed, the R-CHOP regimen was switched to the BR regimen based on evidence from a randomized trial implying better 5-year progression-free survival with BR compared with R-CHOP [13]; the BR regimen consisted of bendamustine 96 mg (70% of the standard 90 mg/m^2^ dose) and rituximab 560 mg (375 mg/m^2^). Because her ECOG performance status had deteriorated to 3–4, we prioritized tolerability and avoidance of treatment-related toxicity; therefore, we did not add ibrutinib to BR and administered bendamustine at a reduced dose [4,14]. After removal of both chest tubes, the bilateral pleural effusions resolved, with no re-accumulation thereafter (Figure 1D). Furthermore, generalized lymphadenopathy regressed, and bone marrow examination revealed resolution of lymphoma infiltration, resulting in complete remission (CR).

## 3. Discussion

Our patient developed persistent massive pleural effusions requiring invasive mechanical ventilation and bilateral continuous thoracic drainage. Pleural effusion is an uncommon manifestation of MCL, and it is even more unusual for MCL to present with life-threatening respiratory failure requiring invasive mechanical ventilation and continuous thoracic drainage prior to diagnosis. Accordingly, we conducted a literature review of reported cases of MCL with pleural effusion.

On 5 January 2026, we searched PubMed using the following query: (“mantle cell lymphoma” OR “mantle-cell lymphoma” OR “MCL” OR “Lymphoma, Mantle-Cell” [MeSH]) AND (pleural OR effusion OR hydrothorax OR hemothorax OR chylothorax). This search yielded 132 articles. After reviewing all retrieved articles, we identified reports in which pleural effusion was attributable to MCL based on pleural fluid evaluation, and we summarized eligible articles in Table 1 [15,16,17,18,19,20,21,22,23]. Similarly to our case, pleural effusion most often developed before treatment, and bilateral involvement was the most frequently reported distribution. In terms of the pathophysiology of pleural effusion, all published cases described exudative effusions. Given MCL’s propensity to infiltrate extranodal sites, these effusions are most plausibly attributable to direct pleural infiltration by lymphoma. In our patient, the pleural effusion was also exudative, and MCL cells were identified in the pleural fluid by flow cytometry and on the cell block, supporting direct pleural infiltration as the mechanism of pleural fluid accumulation. Regarding supportive interventions, only one report by Masha et al. described the need for continuous thoracic drainage [19]. To our knowledge, no prior report has documented the use of invasive mechanical ventilation in this context. R-CHOP and BR were the most commonly selected treatment regimens. In the report by Anai et al., concomitant tuberculosis was present and was treated accordingly [22]. Outcomes were poor in the published cases. Of the nine reported patients, five died, and infectious complications accounted for some of these deaths. Death directly attributable to progressive MCL was described in two reports by Safa et al. and Keklik et al., in which R-CHOP failed to control the disease [17,20]. In contrast, although our patient required invasive mechanical ventilation and continuous thoracic drainage, immunochemotherapy rapidly reduced pleural output, leading to durable resolution of pleural effusions and CR. Notably, none of the nine reports provided details on the MCL subtype. Clinically, our patient presented with generalized lymphadenopathy and aggressive, life-threatening pleural effusions, which are more compatible with cMCL. However, cMCL frequently exhibits a complex karyotype, whereas our patient had a normal karyotype. Further characterization using comprehensive genomic profiling, such as whole-genome sequencing (WGS), may be required to clarify the underlying biology in such cases. Accumulation of additional cases with integrated genomic data will be important to better define the clinical course and prognosis of MCL complicated by pleural effusion.

In contrast, pleural effusion is relatively common in aggressive B-cell lymphomas such as diffuse large B-cell lymphoma (DLBCL). In some of these aggressive B-cell lymphomas, aberrant cyclin D1 expression due to *CCND1* rearrangement can occur, making pathological differentiation from MCL challenging [1,24]. In such situations, CD5 and SOX11 are useful discriminatory markers; their co-expression strongly favors MCL over cyclin D1–positive aggressive B-cell lymphomas [2,24]. In our patient, the lymphoma cells were positive for cyclin D1, CD5, and SOX11, thereby firmly supporting the diagnosis of MCL rather than cyclin D1-positive DLBCL despite the aggressive clinical presentation with massive pleural effusions.

Several biological features may be relevant to the aggressive clinical behavior. BCL6-positive MCL has been reported to be associated with an unfavorable prognosis [25]. Our patient’s MCL was BCL6-positive, which may, at least in part, explain the aggressive clinical course with pleural effusion. In addition, *TP53* aberrations, including *TP53* mutations and deletions, are regarded as the most robust molecular prognosticators and are associated with treatment resistance and poor outcomes [26]. Although p53 immunohistochemistry was negative in our case, truncating *TP53* variants may not be detected by immunohistochemistry [4]. Therefore, assessment by DNA sequencing would be preferable to more definitively evaluate *TP53* status. Beyond these immunohistochemical markers, genomic complexity, defined as a complex karyotype on conventional cytogenetics or more than three copy-number variations (CNVs), has emerged as an independent poor prognostic marker in patients treated with either immunochemotherapy or BTK inhibitors, and these findings have been replicated using WGS [2,4]. Although WGS was not performed in our patient, the severity of presentation raises the possibility of underlying genomic complexity.

To date, no dedicated studies have addressed whether MCL with pleural effusion has a worse prognosis than MCL without pleural effusion. However, several reports indicate that malignant lymphoma presenting with pleural effusion at initial diagnosis is associated with an adverse prognosis [27]. In DLBCL complicated by serous effusions, including pleural effusion, the presence of malignant effusion has been identified as an independent poor prognostic factor in multivariate analysis [28]. Moreover, a baseline pleural effusion volume ≥200 mL has been reported to correlate with inferior survival [29]. In our patient, the malignant pleural effusion clearly exceeded this threshold at presentation, and thoracic drainage consistently yielded more than 1 L/day. Although MIPI and MIPI-c were used for risk stratification in MCL, neither index explicitly incorporates pleural effusions. Whether pleural effusion represents an independent adverse prognostic marker in MCL remains unknown because dedicated cohorts are lacking. Nevertheless, by cautious analogy to DLBCL, malignant pleural effusions may identify a biologically aggressive subset with higher tumor burden and extranodal spread in MCL. This prognostic reasoning directly influenced our clinical decision-making, such as early initiation of prephase prednisone and R-CHOP without waiting for final histopathology. Afterward, we switched to BR, which has been associated with more favorable 5-year progression-free survival [13]. However, in a younger and fitter patient, the addition of a BTK inhibitor, such as ibrutinib or acalabrutinib, to R-CHOP or BR would also be a reasonable therapeutic option [4,14].

Given the rarity of this aggressive clinical course and her unfavorable risk stratification by the MIPI (high risk) and the MIPI-c (high-intermediate risk), relapse remains a plausible concern. Nevertheless, treatment options for relapsed or refractory (R/R) MCL have advanced substantially in recent years. Anti-CD19 chimeric antigen receptor (CAR) T-cell therapy has emerged as a highly active salvage option for R/R MCL. In ZUMA-2 cohort 3, brexucabtagene autoleucel achieved a 91% overall response rate with a 73% CR rate, with favorable 12-month estimates for progression-free and overall survival [30]. Likewise, lisocabtagene maraleucel demonstrated durable remissions in the TRANSCEND NHL 001 MCL cohort [31]. In parallel, the CD20 × CD3 T-cell–engaging bispecific antibody glofitamab has also shown promising efficacy in R/R MCL [32]. If our patient were to relapse, these novel therapies could still offer clinically meaningful benefit and represent important salvage options.

Despite these aggressive features, our patient achieved CR. We attribute this favorable outcome to early initiation of prephase prednisone and prompt introduction of immunochemotherapy with R-CHOP, followed by BR after the diagnosis of MCL was confirmed. This treatment strategy effectively reduced pleural output, allowed discontinuation of mechanical ventilation and thoracic drainage, and ultimately led to CR. Therefore, our case illustrates that even in de novo MCL presenting with massive pleural effusions and respiratory failure, a combination of appropriate ventilatory management, timely thoracic drainage, and rapid initiation of immunochemotherapy can be both feasible and potentially lifesaving. This case was also shared in our regular institutional educational conference, reinforcing its educational value and emphasizing the importance of timely diagnosis with appropriate treatment in similar presentations.

Because large-scale data specifically focusing on MCL with pleural effusion are lacking, the long-term risk of relapse and overall prognosis in such cases remain uncertain. Accumulation of additional cases and larger cohort studies is needed to clarify the prognostic impact of pleural effusion in MCL and to optimize treatment strategies for this rare but clinically challenging presentation.

## 4. Conclusions

De novo MCL complicated by persistent massive pleural effusions requiring invasive mechanical ventilation and continuous thoracic drainage before diagnosis is rare. However, in critically ill patients with rapidly progressive and otherwise unexplained massive pleural effusions, malignant lymphoma should be considered early, and prompt diagnostic evaluation should be pursued. An integrated approach comprising a thorough diagnostic workup, appropriate ventilatory management, timely thoracic drainage, and prompt initiation of immunochemotherapy can reduce pleural output, enable extubation, and be lifesaving. Clinicians should recognize that MCL rarely presents with persistent massive pleural effusions.

## Figures and Tables

**Table 1:** Literature review of mantle cell lymphoma with pleural effusion.

Author	Age	Sex	Onset	Laterality	Fluid Type	Interventions	Treatment	Outcome
Desai [15]	71	Female	Pre-treatment	Right	NR	Thoracocentesis	R-cytarabine → BR	Alive
Obregon [16]	73	Female	Pre-treatment	Left	Exudate	Thoracocentesis	BR	Alive
Safa [17]	69	Male	Relapse	Left	NR	Thoracocentesis	R-CHOP	Dead
Kai [18]	71	Male	Pre-treatment	Bilateral	NR	Thoracocentesis	CHOP → BR	Dead
Masha [19]	70	Male	Pre-treatment	Right	Exudate	Thoracic drainage	R-CHOP	Dead
Keklik [20]	55	Female	Pre-treatment	Bilateral	Exudate	Thoracocentesis	R-CHOP	Dead
Yonal [21]	75	Male	Pre-treatment	Bilateral	Exudate	Thoracocentesis	CHOP	Dead
Anai [22]	78	Female	Refractory	Right	Exudate	Thoracocentesis	Anti-TB	Alive
Thompson [23]	97	Male	Pre-treatment	Bilateral	NR	Thoracocentesis	BSC	Alive

Abbreviations: NR, not reported; R, rituximab; BR, bendamustine and rituximab; R-CHOP, rituximab, cyclophosphamide, doxorubicin, vincristine, and prednisone; TB, tuberculosis; BSC, best supportive care.

## Data Availability

The original data presented in the study are included in the article, further inquiries can be directed to the corresponding author.

## Abbreviations

The following abbreviations are used in this manuscript:

MCL	Mantle cell lymphoma
R-CHOP	Rituximab, cyclophosphamide, doxorubicin, vincristine, and prednisone
BR	Bendamustine and rituximab
CT	Computed tomography

## References

[1] López C., Silkenstedt E., Dreyling M., Beà S. (2024). Biological and clinical determinants shaping heterogeneity in mantle cell lymphoma. Blood Adv..

[2] Campo E., Jaffe E.S., Cook J.R., Quintanilla-Martinez L., Swerdlow S.H., Anderson K.C., Brousset P., Cerroni L., de Leval L., Dirnhofer S. (2022). The International Consensus Classification of Mature Lymphoid Neoplasms: A report from the Clinical Advisory Committee. Blood.

[3] Silkenstedt E., Dreyling M. (2023). Mantle cell lymphoma-Update on molecular biology, prognostication and treatment approaches. Hematol. Oncol..

[4] Jerkeman M., Aurer I., Campo E., Cheah C.Y., Clark J., Doorduijn J., Eyre T.A., Fehr M., Giné E., Gomes da Silva M. (2025). EHA-EU MCL network guidelines for diagnosis and treatment of mantle cell lymphoma. HemaSphere.

[5] Romaguera J.E., Medeiros L.J., Hagemeister F.B., Fayad L.E., Rodriguez M.A., Pro B., Younes A., McLaughlin P., Goy A., Sarris A.H. (2003). Frequency of gastrointestinal involvement and its clinical significance in mantle cell lymphoma. Cancer.

[6] Argatoff L.H., Connors J.M., Klasa R.J., Horsman D.E., Gascoyne R.D. (1997). Mantle cell lymphoma: A clinicopathologic study of 80 cases. Blood.

[7] Baheti A.D., Tirumani S.H., Sewatkar R., Sachin S.S., Shinagare A.B., Ramaiya N.H. (2015). MDCT of extranodal mantle cell lymphoma: A single institute experience. Abdom. Imaging.

[8] Vega F., Padula A., Valbuena J.R., Stancu M., Jones D., Medeiros L.J. (2006). Lymphomas involving the pleura: A clinicopathologic study of 34 cases diagnosed by pleural biopsy. Arch. Pathol. Lab. Med..

[9] Jain P., Wang M. (2019). Mantle cell lymphoma: 2019 update on the diagnosis, pathogenesis, prognostication, and management. Am. J. Hematol..

[10] Nadeu F., Martin-Garcia D., Clot G., Díaz-Navarro A., Duran-Ferrer M., Navarro A., Vilarrasa-Blasi R., Kulis M., Royo R., Gutiérrez-Abril J. (2020). Genomic and epigenomic insights into the origin, pathogenesis, and clinical behavior of mantle cell lymphoma subtypes. Blood.

[11] Hoster E., Dreyling M., Klapper W., Gisselbrecht C., van Hoof A., Kluin-Nelemans H.C., Pfreundschuh M., Reiser M., Metzner B., Einsele H. (2008). A new prognostic index (MIPI) for patients with advanced-stage mantle cell lymphoma. Blood.

[12] Hoster E., Rosenwald A., Berger F., Bernd H.W., Hartmann S., Loddenkemper C., Barth T.F., Brousse N., Pileri S., Rymkiewicz G. (2016). Prognostic Value of Ki-67 Index, Cytology, and Growth Pattern in Mantle-Cell Lymphoma: Results From Randomized Trials of the European Mantle Cell Lymphoma Network. J. Clin. Oncol..

[13] Flinn I.W., van der Jagt R., Kahl B., Wood P., Hawkins T., MacDonald D., Simpson D., Kolibaba K., Issa S., Chang J. (2019). First-Line Treatment of Patients With Indolent Non-Hodgkin Lymphoma or Mantle-Cell Lymphoma With Bendamustine Plus Rituximab Versus R-CHOP or R-CVP: Results of the BRIGHT 5-Year Follow-Up Study. J. Clin. Oncol..

[14] Wang M.L., Jurczak W., Jerkeman M., Trotman J., Zinzani P.L., Belada D., Boccomini C., Flinn I.W., Giri P., Goy A. (2022). Ibrutinib plus Bendamustine and Rituximab in Untreated Mantle-Cell Lymphoma. N. Engl. J. Med..

[15] Desai R., Khazey K., Sandhu H., Makar P., Randhawa N., Khalyfa A., Khan M., Yarbrough A., Spyratos T. (2024). Gastrointestinal Symptom-Free Multiple Lymphomatous Polyposis: An Atypical Case Presentation of Mantle Cell Lymphoma. Case Rep. Gastroenterol..

[16] Obregon M., Kohli A., Song M. (2023). Mantle Cell Lymphoma Causing Recurrent Pleural Effusions: A Case Report. Cureus.

[17] Safa F., Rasmussen T., Lobelle-Rich P., Collier S., Milligan N., Schmeig J., Schmid J., Wiewiorowski C., Totaro D., Brown T.C. (2022). Establishment and characterization of a new mantle cell lymphoma cell line with a NOTCH2 mutation. Arbo. eJHaem.

[18] Kai K., Ryu Y., Kamochi K., Nishioka A., Kubota Y., Nakamura M., Kimura S., Sueoka E., Aishima S. (2018). Synchronous mantle cell lymphoma and lung adenocarcinoma presenting in a pleural effusion: A rare tumour combination and a potential pitfall of cytodiagnosis. Cytopathology.

[19] Masha L., Zinchuk A., Boosalis V. (2015). Synchronous Pulmonary Malignancies: Atypical Presentation of Mantle Cell Lymphoma Masking a Lung Malignancy. Rare Tumors.

[20] Keklik M., Yildirim A., Keklik E., Ertan S., Deniz K., Ozturk F., Ileri I., Cerci I., Camlica D., Cetin M. (2015). Pericardial, pleural and peritoneal involvement in a patient with primary gastric mantle cell lymphoma. Scott. Med. J..

[21] Yonal I., Ciftcibasi A., Gokturk S., Yenerel M.N., Akyuz F., Karaca C., Demir K., Besisik F., Kalayoglu-Besisik S. (2012). Massive ascites as the initial manifestation of mantle cell lymphoma: A challenge for the gastroenterologist. Case Rep. Gastroenterol..

[22] Anai S., Hashisako M., Ikegame S., Wakamatsu K., Nagata N., Nakanishi Y., Kajiki A. (2012). Mantle cell lymphoma involvement of the pleura and tuberculous pleurisy with pulmonary tuberculosis: A case report and literature review. J. Infect. Chemother..

[23] Thompson B., Gonzalez M.R. (2010). Ninety-seven Years Old Hispanic Male With Mantle Cell Lymphoma. J. Clin. Med. Res..

[24] Cheng J., Hashem M.A., Barabé F., Cloutier S., Xi L., Raffeld M., Pittaluga S., Jaffe E.S. (2020). CCND1 Genomic Rearrangement as a Secondary Event in High Grade B-Cell Lymphoma. HemaSphere.

[25] Castillo D.R., Park D., Jeon W.J., Joung B., Lee J., Yang C., Pham B., Hino C., Chong E., Shields A. (2023). Unveiling the Prognostic Significance of BCL6+/CD10+ Mantle Cell Lymphoma: Meta-Analysis of Individual Patients and Systematic Review. Int. J. Mol. Sci..

[26] Loh Z., Yeh P., Keane C., Hawkes E.A. (2025). Advances in biomarkers for mantle cell lymphoma in the era of targeted therapies. Haematologica.

[27] Das D.K. (2006). Serous effusions in malignant lymphomas: A review. Diagn. Cytopathol..

[28] Chen Y.P., Huang H.Y., Lin K.P., Medeiros L.J., Chen T.Y., Chang K.C. (2015). Malignant effusions correlate with poorer prognosis in patients with diffuse large B-cell lymphoma. Am. J. Clin. Pathol..

[29] Porcel J.M., Cuadrat I., García-Cerecedo T., Pardina M., Bielsa S. (2019). Pleural Effusions in Diffuse Large B-Cell Lymphoma: Clinical and Prognostic Significance. Lung.

[30] van Meerten T., Kersten M.J., Iacoboni G., Hess G.R., Mutsaers P.G.N.J., Martin Garcia-Sancho A.M., Goy A., Gine E., Hill B.T., Weng W.K. (2025). Brexucabtagene autoleucel for BTKi-naive relapsed/refractory mantle cell lymphoma: Primary analysis of ZUMA-2 Cohort 3. Blood.

[31] Abramson J.S., Palomba M.L., Gordon L.I., Lunning M., Wang M., Arnason J., Purev E., Maloney D.G., Andreadis C., Sehgal A. (2024). Two-year follow-up of lisocabtagene maraleucel in relapsed or refractory large B-cell lymphoma in TRANSCEND NHL 001. Blood.

[32] Phillips T.J., Carlo-Stella C., Morschhauser F., Bachy E., Crump M., Trněný M., Bartlett N.L., Zaucha J., Wrobel T., Offner F. (2025). Glofitamab in Relapsed/Refractory Mantle Cell Lymphoma: Results From a Phase I/II Study. J. Clin. Oncol..

